# Potassium

**DOI:** 10.1016/j.advnut.2023.06.004

**Published:** 2023-06-10

**Authors:** Lynda A. Frassetto, Almudena Goas, Richard Gannon, Susan A. Lanham-New, Helen Lambert

**Affiliations:** 1 Department of Medicine/Division of Nephrology, University of California San Francisco, CA, USA; 2 Department of Medicine, Universidad Francisco de Vitoria, Madrid, Spain; 3 School of Medicine, Medical Sciences & Nutrition, Forester hill, University of Aberdeen, Aberdeen, United Kingdom; 4 Department of Nutrition, Food & Exercise Sciences, Faculty of Health and Medical Sciences, University of Surrey, Guildford, Surrey, United Kingdom

## Introduction

Potassium is the main intracellular cation in the body and is principally involved in membrane potential and electrical excitation of both nerve and muscle cells as well as acid–base regulation. The compartmentalization of potassium is predominantly maintained by the (energy-dependent) cellular uptake of the element and the concomitant extrusion of sodium by the specific cell membrane-bound enzyme, sodium–potassium adenosine triphosphatase.

On average, the potassium content of an adult human is estimated to be ∼40 to 50 mmol (1.6–2.0 g)/kg body weight, so a 70-kg adult would have ∼2800 to 3500 mmol of potassium (110–137 g). Intracellular potassium concentration is 150 mmol/L (5.9 g/L), with the remainder present in the extracellular fluid. The extracellular fluid concentration of potassium is rather strictly maintained at a concentration of 3.5–5.5 mmol/L (137–215 mg/L). The total body potassium reflects lean tissue mass because ∼90% to 95% of this potassium is intracellular in the muscle and bone; thus, there is variation according to the degree of the body’s muscularity [1].

Ingested potassium is mainly excreted in the urine (∼80% to 90%). The remaining 10%–20% is excreted in feces and sweat. Most of the potassium that is filtered by the kidney glomerulus is then reabsorbed throughout the kidney tubules. High-extracellular potassium concentrations stimulate the release of aldosterone, which then promotes increased distal tubular secretion of potassium into the urine. Blood potassium concentrations are also independently under circadian rhythm control so that dietary potassium excretion is greater during daylight hours than at night.

## Deficiencies

A potassium deficiency of nutritional origin is uncommon because potassium is widely available in several foods. However, due to the extensive consumption of processed foods, significant percentages of populations in Westernized regions may have discrete but (very) relevant potassium deficiency. This could result in moderate chronic total body potassium depletion [2].

## Diet recommendations

Requirements for dietary potassium intake are difficult to determine precisely, and this is reflected in the wide recommendation differences that exist between countries and authorities like the WHO or European Food Safety Authority. They can be estimated from the amount of potassium that is accumulated with growth as well as from urinary and fecal excretion. Mandatory potassium loss from the kidneys is ∼10 to 15 mmol/L of urine (that is, the kidneys cannot make the urine potassium content more concentrated). Fecal excretion of potassium may represent homeostatic excretion of excessive intakes or losses incurred in maintaining sodium homeostasis. The amount of potassium required for both growth and the synthesis of lean tissue has been determined to be 50 mmol/L (2.0 g/kg body weight) in the United Kingdom. The United States adequate intake, the UK lower reference nutrient intake and reference nutrient intake, and the European Union and WHO recommendations are shown in Table 1. For adult populations (>18 y), the United States adequate intake is higher for men than that for women, 3.4 compared with 2.6 g, and higher during pregnancy and lactation. The UK reference nutrient intake is 3.5 g. The European Union population reference intake for potassium is 3.1 g/d, and the lowest threshold intake is 1.6 g/d. The most recent WHO recommendation for adults >16 y is 3.5 g.

**TABLE 1:** Dietary recommendations for potassium^1^

	Sex	US AI	UK LRNI	UK RNI	EFSA AI	WHO
**0–3 mo**	**—**	**—**	400	800	**—**	**—**
**0–6 mo**	**—**	400	**—**	**—**	**—**	**—**
**4–6 mo**	**—**	**—**	400	850	**—**	**—**
**7–9 mo**	**—**	**—**	400	700	**—**	**—**
**7–12 mo**	**—**	860	**—**	700	750	**—**
**0–12 mo**	**—**	**–**	450	**—**	**—**	**—**
**1–3 y**	**—**	2000	450	800	800	**—**
**2–15 y**	**—**	**—**	**—**	**—**	**—**	Adjusted downward from adult values, dependent on energy requirements for age
**4–6 y**	**—**	**—**	600	1100	1100	**—**
**4–8 y**	**—**	2300	**—**	**—**	**—**	**—**
**7–10 y**	**—**	**—**	950	2000	1800	**—**
**9–13 y**	**—**	2500	1600	3100	2700	**—**
**11–14 y**	**—**	2300	**—**	**—**	**—**	**—**
**14–18 y**	**male**	3000	**—**	**—**	**—**	**—**
**–**	**female**	2300	**—**	**—**	**—**	**—**
**–**	**pregnancy**	2600	**—**	**—**	**—**	**—**
**–**	**lactation**	2500	**—**	**—**	**—**	**—**
**15–17 y**	**—**	**—**	**—**	**—**	3500	**—**
**15–18 y**	**—**	**—**	2000	3500	**—**	**—**
**>16 y**	**—**	**—**	**—**	**—**	**—**	3510
**19–50 y**	**male**	3400	2000	3500	3500	**—**
**–**	**female**	2600	**—**	**—**	**—**	**—**
**–**	**pregnancy**	2900	**—**	**—**	3500	**—**
**–**	**lactation**	2800	**—**	**—**	3500	**—**
**51+ y**	**male**	3400	**—**	**—**	**—**	**—**
**–**	**female**	2600	**—**	**—**	**—**	**—**

US	https://ods.od.nih.gov/factsheets/Potassium-HealthProfessional/#en11
UK (not changed since 1991)	https://assets.publishing.service.gov.uk/government/uploads/system/uploads/attachment_data/file/618167/government_dietary_recommendations.pdf
WHO 2012	file:///C:/Users/hl0012/OneDrive%20-%20University%20of%20Surrey/Documents/potassium%20WHO%202012.pdf
EFSA 2016	https://efsa.onlinelibrary.wiley.com/doi/10.2903/j.efsa.2016.4592

1 AI, adequate intake; EFSA, European Food Safety Authority; LRNI, lower reference nutrient intake; RNI, reference nutrient intake; WHO, World Health Organization.

## Food sources

Not surprisingly, because potassium is a main intracellular ion, potassium in the diet is derived from a wide variety of sources. Potatoes, soft drinks, meat and meat products, cereal and cereal products, and milk and milk products each provide >10% of the potassium by energy intake in United States and UK population groups (Table 2). For milk and milk products, approximately half (6%) comes from semi-skimmed milk. Vegetables, fruits, and nuts provide >5% of potassium intake in the diet. Bananas and tropical fruits are a rich source of potassium, as are leafy green vegetables and root vegetables (Table 2). Dietary supplements make a negligible contribution to mean potassium intake in the United States, United Kingdom, and European Union. It is very important to note that when potassium is added as a preservative during processing, it is usually as potassium chloride (an acid salt), whereas it is usually present in fruit and vegetables as potassium citrate (an alkali salt). Approximately 85% of dietary potassium intake is absorbed in the gastrointestinal tract, and it is generally considered that the healthiest way to increase potassium intake is to consume more fruit and vegetables.

**TABLE 2:** Contribution of foods to dietary potassium (K) intake Potassium content of foods in selected foods in these categories(Listed in order of contribution to potassium intake)

Food group	Contribution to energy intake (% energy)		Contribution to K intake (% of K intake)^2^	K content of typical foods^1^		
	UK^2^	US^3^			mg/100 g	mg/portion^4^
**Potatoes and potato products**	5	**—**	16	*Chips (fries)*	544	598
				*Potato (baked)*	360	648
**Meat and meat products**	15	9	15	*Chicken breast (grilled)*	460	598
				*Beef burger (grilled)*	270	108
**Cereals and cereal products**	32	27	15	*Bread wholemeal*	253	91
				*Pasta (white, boiled)*	114	262
**Milk and milk products**	11	11	13	*Milk (semi-skimmed)*	152	304
				*Cheese (hard)*	77	46
**Fruit (including juice, dried fruit), nuts and seeds**	6	10	12	*Bananas*	330	330
				*Apples*	100	100
**Vegetables (including pulses)**	5	6	11	*Spinach (baby, boiled)*	950	760
				*Avocado*	430	602
**Beverages (including tea, coffee, alcohol)**	7	**—**	8	*Coffee with milk*	97	252
				*Beer (lager)*	39	224
**Fish and fish dishes**	4	**—**	4	*Cod (grilled)*	453	544
				*Tuna (canned)*	230	212
**Eggs and egg dishes**	3	**—**	2	*Eggs*	145	87
**Confectionery, sugar, and preserves**	2	**—**	1	*Chocolate (plain)*	300	150
**Savory snack**	1	**—**	1	*Crisps*	1328	359

Abbreviations: MAFF, Ministry of Agriculture, Fisheries and Food; NHANES, National Health and Nutrition Examination Survey.

1 McCance and Widdowson Composition of Foods Integrated Dataset 2021

2 UK National Diet and Nutrition Survey Rolling Programme Years 9–11 (2016/17–2018/19)

3 NHANES III, https://wwwn.cdc.gov/nchs/nhanes/nhanes3/default.aspx

4 Average portion sizes from MAFF Food portion sizes 1988, and retail sources.

Current dietary trends in Western populations show a consistent trend for decreased consumption of fruit and vegetables and increased intake of sodium-rich foods. This results in reduced potassium intake and increased sodium intake; these ions have separate pathophysiologic consequences, and the higher the sodium intake, the greater the urinary potassium loss, so that the ratio of sodium:potassium intake may be as important as potassium intake alone.

## Clinical use of potassium

A high-potassium intake has been shown to have protective effects against a number of pathologic states that affect the cardiovascular system, kidneys, and musculoskeletal health. Increased potassium intake lowers blood pressure, and this effect has been consistent in both hypertensive and normotensive populations. Evidence suggests that potassium may be effective in reducing stroke and could help prevent chronic kidney damage. Increasing daily alkali intake, which is linked to potassium (found in fruits and vegetables), helps to reduce calcium excretion in the urine and thus may have a positive effect on musculoskeletal health. There is also growing evidence of a strong link between increasing potassium alkali intake and favorable effects on muscle function, overall muscle health, and potentially, prevention of falls. However, these effects cannot be attributed to the action of potassium independent of the alkali intake.

## Toxicity

Healthy individuals ingesting the usual dietary intakes of potassium are unlikely to have problems with potassium toxicity. Potassium intake in Western societies is normally in the range of 1.6–5.9 g/d. Extremely high intakes of potassium >17.6 g/d (usually only obtained with potassium supplements) have been associated with symptomatic hyperkalemia. High-blood concentrations of potassium can cause muscle weakness and cardiac arrhythmias. Chronic damage to the kidneys and some kinds of medications (eg, those used for blood pressure control that block the renin–angiotensin system) can cause high-potassium levels if dietary potassium intake is not controlled.

## Recent research

Dietary potassium intake has been shown to significantly lower blood pressure. Studies have demonstrated that this occurs in a dose-responsive manner in hypertensive and nonhypertensive patients, with evidence coming from a number of important observational studies and clinical trials. Several key meta-analyses also support this finding. In particular, the DASH studies demonstrated that increasing potassium intake and reducing sodium intake are additive in lowering blood pressure [3]. Potassium-induced reductions in blood pressure significantly lower the incidence of a cerebrovascular accident (stroke), CHD, MI, and other cardiovascular events [4]. There are also data to show that potassium reduces the risk of cerebrovascular accidents independent of blood pressure [4].

Increasing potassium alkali intake has also been shown to conclusively reduce urinary calcium excretion, thus creating a positive calcium balance. In the longer term, this is likely to have very beneficial effects on skeletal health and the concomitant risk of osteoporosis. Clinical trials support these findings in the short term (3–6 mo), with a number of important randomized, controlled trials showing a reduction in bone resorption markers in those individuals on potassium citrate/potassium bicarbonate supplementation/high dietary potassium intake [5,6]. Further longer term studies (>12 mo) evaluating the effects of high dietary potassium and calcium intake on blood pressure, urinary calcium excretion, and bone resorption are now urgently required.

Increasing potassium alkali intake has also been shown to reduce the risk of kidney stones. Interestingly, studies in hypertensive rats show that high-potassium intake prevents renal vascular, glomerular, and tubular damage independent of blood pressure. Currently, in humans there is no direct evidence that potassium protects against renal arteriolar/tubular lesions that specifically occur in kidney disease or hypertension [7].

There is also recent interest in being able to increase fruit and vegetable intake in subjects with moderate to severe kidney failure [8]. One new option is the availability of oral potassium binders, which would prevent excessive uptake of potassium but would permit ingestion of other important dietary factors, such as trace minerals, fiber, and antioxidants.

Finally, the transition toward the Westernized diet has led to populations consuming substantially less potassium compared with dietary intakes in preagricultural times, when humans consumed a diet high in potassium (>200 mmol/d). With the increase in consumption of processed foods and the parallel reductions (often dramatic) in fruits and vegetables, potassium intakes are <50 mmol/L in large numbers of societies. These reductions are, in many populations worldwide, also mirrored by high-sodium intakes. How such dietary and nutrient intake trends can be reversed is an important social and medical question.

### Conflict of interest

The authors report no conflicts of interest.

### Funding

The authors reported no funding received for this study.
